# Collective plasticity of binocular interactions in the adult visual system

**DOI:** 10.1038/s41598-024-57276-8

**Published:** 2024-05-07

**Authors:** Mengxin Wang, Paul V. McGraw, Timothy Ledgeway

**Affiliations:** School of Psychology, University of Nottingham, Nottingham, NG7 2RD UK

**Keywords:** Psychology, Visual system, Attention, Perception

## Abstract

Binocular visual plasticity can be initiated via either bottom-up or top-down mechanisms, but it is unknown if these two forms of adult plasticity can be independently combined. In seven participants with normal binocular vision, sensory eye dominance was assessed using a binocular rivalry task, before and after a period of monocular deprivation and with and without selective attention directed towards one eye. On each trial, participants reported the dominant monocular target and the inter-ocular contrast difference between the stimuli was systematically altered to obtain estimates of ocular dominance. We found that both monocular light- and pattern-deprivation shifted dominance in favour of the deprived eye. However, this shift was completely counteracted if the non-deprived eye’s stimulus was selectively attended. These results reveal that shifts in ocular dominance, driven by bottom-up and top-down selection, appear to act independently to regulate the relative contrast gain between the two eyes.

## Introduction

Recent evidence has revealed surprising levels of neural plasticity in mechanisms governing sensory eye dominance, even in human adults. The relative contribution an eye makes to binocular perception can be enhanced after just several hours of modified input, accomplished by means such as light or pattern deprivation, while leaving the input to the other eye unaffected^1–3^. The fact that the shift in eye dominance is associated with an increase in perceived contrast of the stimulus in the treated eye, suggests that altered contrast gain control may underlie the effect^4^. Follow on work, using ultra-high-field magnetic resonance spectroscopy in humans, has revealed associated changes in resting GABA level in primary visual cortex (V1), alongside the perceptual enhancement of the deprived eye. The decrease in resting GABA following monocular deprivation, is thought to trigger homeostatic plasticity in V1 enabling the subsequent shift in eye dominance^5^. In adult primates, intrinsic optical imaging of signals from ocular dominance columns in V1 show a similar pattern, where the contribution of the non-deprived eye steadily decreases while the other eye is deprived, leading to an elevated contribution of the deprived eye once the patch is removed^6^. This implies a low-level cortical mechanism (V1) that sets the balance of ocular dominance based on the recent sensory history of each eye.

Wang et al.^7^ have recently shown that depriving one eye of light, or spatial input, is not a pre-requisite for generating shifts in eye dominance. Three different manipulations of monocular visual input—light deprivation, spatial deprivation or image inversion via a prism—produced similar shifts in eye dominance measured using binocular rivalry, favouring the treated eye. Importantly, the prism condition preserved the luminance and contrast information in the treated eye. Further, the degree of shift in eye dominance was regulated by the attentional demands imposed during the monocular treatment period^7^. This result suggests an alternative mechanism, based on attentional eye selection, that can also drive shifts in eye dominance.

The prism effects are consistent with the notion that sensory eye dominance during binocular rivalry can be modulated by top-down attention—perception is biased towards one stimulus when attention is selectively directed to that eye^8,9^. Crucially though, the timescale and direction of the shift in eye dominance differ between these situations. In the case of changes in selective attention *during* binocular rivalry, the effects are instantaneous and it is the attended eye that dominates perception for longer periods^10^. Whereas, following short-term manipulation of monocular input, the changes in eye dominance occur after normal binocular vision is restored and favour the eye that was not selectively attended to^7^. Several different methods have been used in experiments to direct top-down attention selectively to one of the two eyes. In some studies, participants performed a task requiring consistent attention to one eye's stimulus but not the other^8,9^. For example, Chong et al.^9^ asked participants to count the frequency of changes in features (e.g. spatial frequency) of a named stimulus, whilst subjective visual reports were tracked during binocular rivalry. In other studies, the engagement of selective attention has relied on explicit attentional instructions^10,11^.

There is strong empirical evidence that both bottom-up and top-down regulation of sensory eye dominance occurs. However, it is unknown if these mechanisms interact with one another. Here, we sought to address this issue by first quantifying the effects of (top-down) selective attention on sensory eye dominance measured with a binocular rivalry task, and then examining whether these effects could modulate the eye dominance shifts observed following (bottom-up) short-term manipulation of the input to one eye.

## Methods

### Observers

A convenience sample of seven observers volunteered to participate in this study (age range: 24–50 years, three females), who were staff members or students of the University of Nottingham. Based on the effect sizes (average *d* = 2.63 when measured immediately following the removal of eye patch) reported by a previous study employing similar protocols of short-term monocular deprivation^7^, a sample size of 3, calculated using G*Power 3.1^12^, was required to achieve a power more than 80% to detect expected changes in eye dominance. In addition, a sample size of 4 was sufficient to observe reliable effects of endogenous attention on perceptual dominance during binocular rivalry^9^.

Five of the participants completed two conditions of monocular deprivation on a randomly chosen eye (fixed for each participant throughout the experiment). First, an opaque eye patch eliminated all visual input to the patched eye. Second, participants wore a pair of goggles where one lens was an optical diffuser that substantially reduced the spatial coherence of light (hereafter referred to as a diffuser). That is, spatial contour information was degraded such that subjects could not, for example, count fingers at a distance of 10 cm, whilst overall luminance was largely preserved (attenuated by ~ 15% as measured by a Minolta CS 110 photometer). We chose to test these two types of monocular deprivation in order to establish whether they have similar or different effects on sensory eye dominance measured using this task. For three of the participants we were able to test a diffuser separately on each eye, as a control to check whether the effects were specific to the eye that was randomly treated. We did this because individuals, even with normal binocular vision, can show large variations in sensory eye dominance^13^ which could influence the pattern of results found. All participants had normal stereo vision (stereoacuity range: 30–60 arcsec) assessed by the TNO stereo test (Laméris Ootech, Nieuwegein, The Netherlands) and normal visual acuity (range: 0–0.16 logMAR on an ETDRS letter chart).

The study was conducted with the approval of the University of Nottingham, School of Psychology Ethics Committee and in accordance with the Declaration of Helsinki. All participants gave informed consent.

### Apparatus and stimuli

The stimulus to each eye was presented on one of a pair of identical LCD monitors (22 inch Samsung Sync-Master 2233RZ; 1024 × 768 pixel resolution; 60 Hz refresh rate; 318 cd/m^2^ maximum luminance). Dichoptic presentation of the stimuli was achieved using a Wheatstone mirror stereoscope, with an optical viewing distance of 231.5 cm. The details of the digital displays used in this experiment have previously been described in^13^.

**Figure 1:**
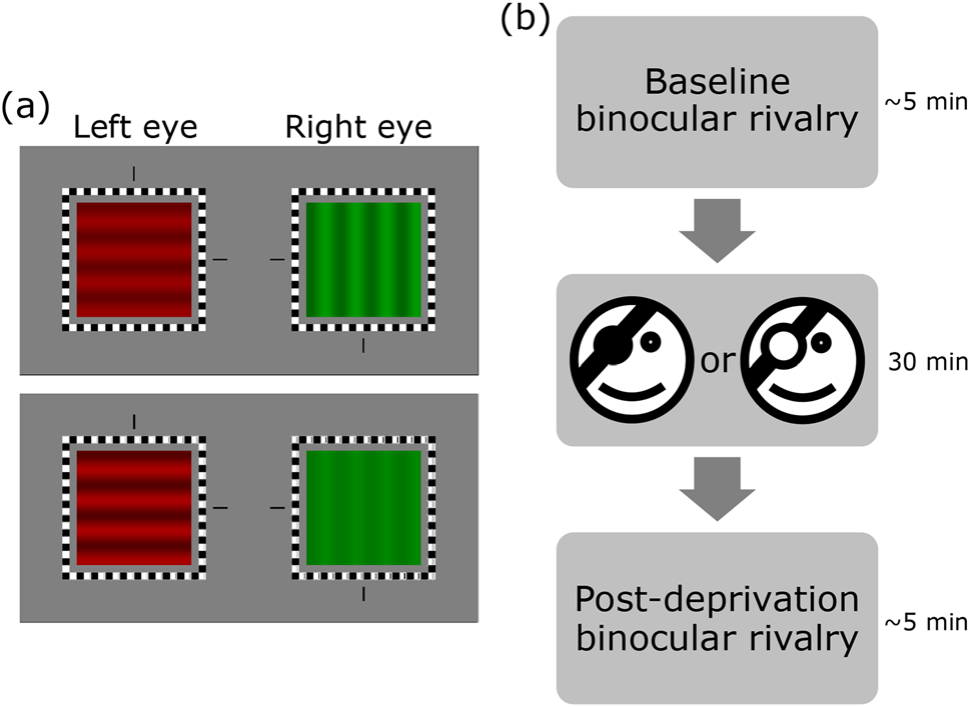
Experimental methods. Example pairs of stimuli used to induce binocular rivalry is shown in (**a**). During a given block of trials, the colour of each eye’s grating was constant, while the orientations were randomly swapped between the two eyes. In the upper panel, the gratings presented to the two eyes have equal contrast; in the lower panel, the left eye’s grating has a higher contrast than that of the right eye’s grating. A schematic illustration of the procedure is shown in (**b**). Perceptual dominance at the onset of binocular rivalry was measured before and after 30-min of monocular deprivation using either an opaque patch or a diffusing lens.

Stimuli were coloured images, generated by an Apple Macintosh computer using custom software written in the C programing language. On the centre of each display, a luminance-modulated sinusoidal grating subtended 2.21° × 2.21° and was framed by a high contrast peripheral fusion lock (alternating black and white square elements). A binocular central fixation cross (presented between trials), and a pair of vertical and horizontal Nonius lines (presented outside the fusion frame on each display) were used to ensure horizontal and vertical fixation disparity was well controlled and to assist binocular fusion (see Fig. 1).

The grating shown to each eye had a spatial frequency of 1.8 cycles per degree and contained 4 full cycles. It was randomly assigned to either + sine or –sine phase on each trial, with respect to the horizontal or vertical midline. The colour of the grating was either red or green, with one colour presented to one eye and the complement to the other, and this arrangement remained fixed during a given run of testing. The pair of gratings presented to the eyes differed not only in colour, but also in orientation: each grating was either horizontal or vertical, randomly chosen on each trial. An example pair of stimuli is shown in Fig. 1.

### Procedure

Sensory eye dominance was measured prior to (baseline) and immediately following a 30-min period monocular deprivation (treatment), delivered via an opaque patch or an optical diffusing lens. Wang et al.^7^ have shown that this period of monocular treatment is sufficient to produce reliable shifts in eye dominance. Figure 1 shows the timeline of the study protocol. It took a minimum of ~ 8 h for each participant to complete the experiment.

Observers were seated in a dimly lit room while performing the task. A chin rest was used to stabilise head position. The mirrors of the stereoscope were placed symmetrically with respect to the median plane of the head, and the angle of each was adjusted for individual observers at the start of each session to ensure stable binocular fusion.

Sensory eye dominance was quantified by measuring the perceptual dominance at onset of binocular rivalry between red and green, orthogonal gratings. An onset rivalry task was chosen because it has the advantage that it enabled us to measure the full psychometric function, and thus comprehensively characterise each participant’s visual performance, across the whole range of conditions tested. The attentional state of the observer was manipulated by either providing no instructions (neutral condition), or by instructing the participant to selectively attend to one of the colours (i.e. red or green) that was tagged to a specific eye (attention condition). For example, when the participant was instructed to “attend to the red grating” and this was presented to the right eye in a given block of trials, it was this eye that was selectively attended (c.f.^14^).

We used a two-alternative forced-choice procedure for the binocular rivalry task. On each trial, the participant pressed a button to trigger the simultaneous grating presentation on each display. The gratings were displayed for a duration of 250 ms. After the gratings were turned off, the participant was required to press one of two buttons to indicate the most dominant orientation perceived on that trial (i.e. either horizontal or vertical). This was then followed by a 1500 ms inter-trial interval. As perceptual dominance during onset binocular rivalry is influenced by inter-ocular contrast differences^15^, within a given run the contrast of the grating viewed by each eye was varied across trials in a yoked manner, using the method of constant stimuli. The Michelson contrast of each grating was varied from 0 to 40% in seven equal steps, constrained by a constant average of 20% (i.e. $\frac{{C}_{RE}+{C}_{LE}}{2}$, where ${C}_{RE}$ and ${C}_{LE}$ denote the contrast of the stimulus presented to the right and the left eyes, respectively). Consequently, the relative contrast, defined as the difference divided by the sum, such as:

1 $$\frac{{C}_{RE}-{C}_{LE}}{{C}_{RE}+{C}_{LE}},$$

in reference to the right eye’s grating, ranged from -1 to 1 and was 0 when the contrast of both gratings was 20%. Each condition of relative contrast was repeated on 20 trials, yielding a total of 140 trials in a run, which lasted approximately 5 min. The colour of the grating presented to each eye was counterbalanced across testing runs for each observer.

There were three test conditions: 1. individual baseline; 2. opaque patch, and 3. diffuser lens. For each condition, at least 6 runs of the binocular rivalry task were completed in a pseudorandom order, including 3 attentional states (i.e. attending to the red grating, attending to the green grating, and no attentional instructions) × 2 eye-colour configurations (i.e. the red grating presented to the right eye and the green grating presented to the left eye, and the converse configuration). The data were collapsed across the 2 eye-colour configurations for each test condition, yielding 3 attention conditions: neutral (no attentional instructions), attending to the deprived eye, and attending to the non-deprived eye. Whenever one of the eye-colour configurations for an attention condition was tested for more than one run, the reverse configuration was tested for the same number of runs, to limit any extraneous bias in perceiving one of the colours. To quantify the dependence of perceptual eye dominance on the relative contrast between the two eyes, a psychometric function was generated for each attention condition for each observer, where each datum was based on at least 40 trials (2 runs × 20 trials).

### Data analyses

**Figure 2:**
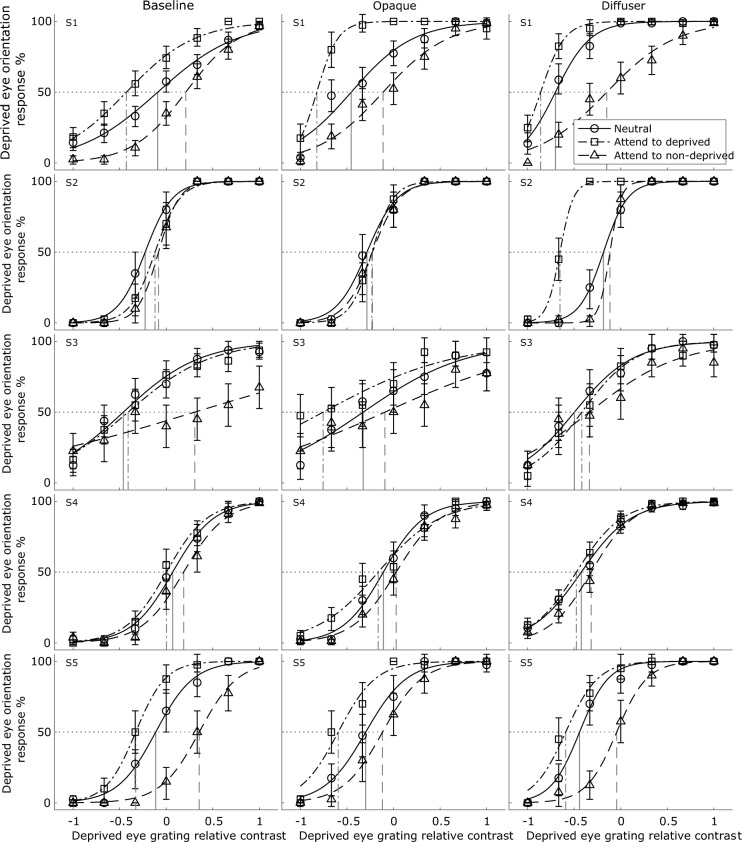
Psychometric functions of the five observers measured in the absence of monocular deprivation (baseline) or following monocular deprivation with an opaque patch or a diffusing lens. The data are plotted in the form of percentage of trials where a dominance of the deprived eye (or to be deprived eye, in the case of the baseline) was reported, as a function of relative contrast of the deprived eye’s grating compared to that presented to the non-deprived eye (calculated in a similar manner as Eq. (1)), in the neutral (circles), attending to the deprived eye (squares), or attending to the non-deprived eye conditions (triangles). The corresponding fitted curves using Eq. (2) are also shown. The abscissae range from -1, indicating zero contrast for the deprived eye’s grating, to 1 where it had maximum contrast. The droplines mark the PSEs for each psychometric function. The error bars represent ± 1 standard error.

**Figure 3:**
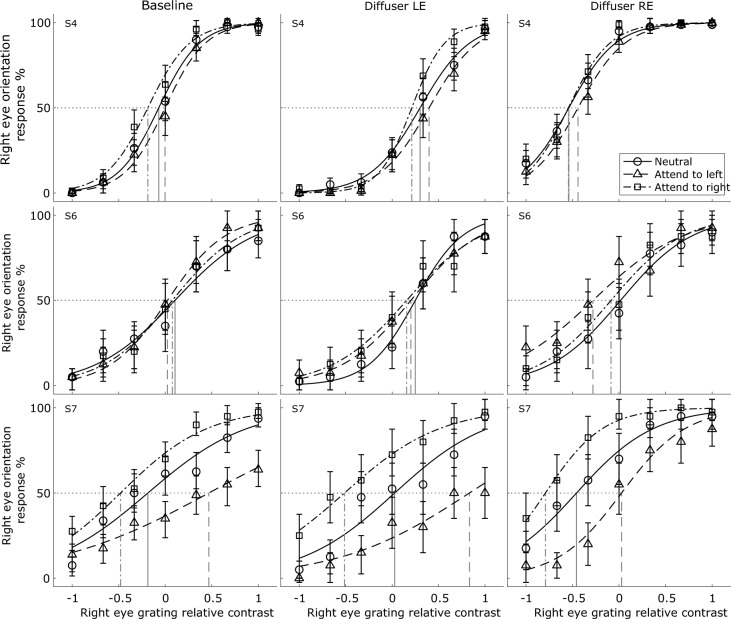
Psychometric functions of the three observers who completed both eye conditions of monocular deprivation using a diffusing lens. All other aspects are the same as in Fig. 2, except that the data are presented in reference to the right eye’s grating for clarity of visualisation.

Psychometric functions for the conditions tested were obtained for each observer, describing the response rate of the grating orientation in either the deprived eye (Fig. 2) or the right eye (Fig. 3), as a function of the relative contrast (in the corresponding eye) for each of the three attentional manipulations (neutral, attend to deprived eye, attend to non-deprived eye).

The data for each condition reported in Fig. 2 are fitted by a 2-parameter logistic function:

2 $$y=\frac{1}{1+{e}^{\frac{-\left(x-b\right)}{a}}}\times 100\mathrm{\%},$$

where $y$ is the percentage of trials on which a dominance of the deprived (*or to be deprived*, in the case of the baseline measurements) eye’s orientation was reported, $x$ is the relative contrast for that grating, $a$ is the slope of the function, and $b$ is the point of subjective equality (PSE) at which both eyes’ gratings were equally likely to be perceived as dominant. The curve fitting was performed using the function *fit* in *Matlab* (MathWorks, Natick, MA), which employed a nonlinear least square method, with the constraint of $-1\le b\le 1$ applied. The fitting details for the data reported in Fig. 3 are the same as in Fig. 2, except that the data are represented in reference to the right eye’s grating.

Statistical analyses were performed to evaluate, at a group level, the shift in PSE and the slope of the psychometric function. To test the effect of deprivation type, data from the five observers were included. To test the effect of left v.s. right eye, data from the three observers were included. Data from all the seven observers were included for the analyses where data were collapsed between the two deprivation type conditions and the two eye conditions.

In addition to the paired-sample t-tests for assessing individual factors, linear mixed-effects model analyses were conducted when interactions were considered. For these models, a full covariance matrix structure was chosen for the random-effects terms. The parameters were estimated with a restricted maximum likelihood method, using the function *fitlme* in MATLAB (MathWorks, Natick, MA).

## Results

We examined sensory eye dominance at the onset of binocular rivalry, before and after short-term monocular deprivation, whilst manipulating the attentional state of observers using explicit instructions. Figure 2 shows psychometric functions with fitted curves for the five observers who completed both conditions of monocular deprivation. Figure 3 shows analogous psychometric functions for the three (control) observers who completed both eye conditions of monocular deprivation using a diffusing lens, to confirm the robustness of the effects.

**Figure 4:**
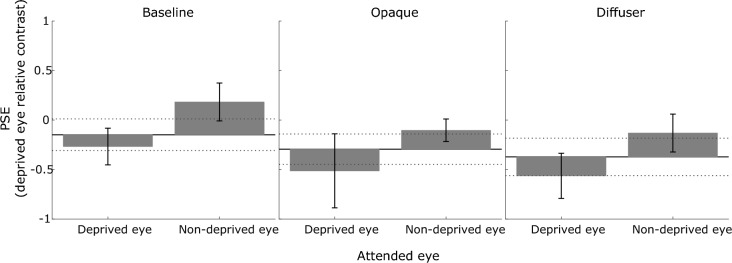
Mean PSE (with respect to the relative contrast of deprived eye’s grating) of the psychometric functions for the seven observers, in the baseline, opaque patching, and diffuser conditions. The horizontal solid line marks the PSE measured in the neutral condition (the dotted lines indicate ± 1 SEM across the group). The bars depict the PSE for attending to the deprived eye and attending to the non-deprived eye conditions, plotted relative to the PSE for the neutral condition. The error bars are ± 1 SEM across the group.

**Figure 5:**
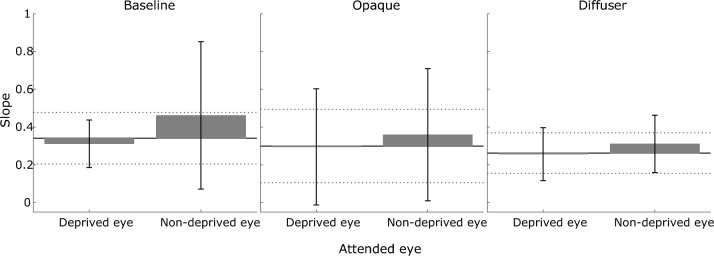
Mean slope of the psychometric functions for the seven observers. Conventions are the same as in Fig. 4.

The estimated parameters from the best-fitting logistic function, averaged across all participants, are summarised in Fig. 4 (*b*, PSE) and Fig. 5 (*a*, slope). For all participants, the relative dominance of an eye increased with the relative contrast of the grating presented to that eye, validating the use of this paradigm to quantify perceptual eye dominance. Notably, some participants showed a baseline bias in eye dominance, reflected by a lateral shift of the psychometric function away from a 0 position on the x-axis, where the gratings in each eye had equal contrast (see Fig. 2, S3, baseline neutral condition).

For baseline measurements without monocular deprivation, providing attentional instructions successfully shifted the psychometric functions laterally, towards the eye presented with a grating of the target colour. This indicates that the attended eye now required less contrast to dominate perception at rivalry onset. This attentional shift was not symmetrical across the eyes for some individuals. For example, the data from S3 (Fig. 2, leftmost plot) show that selectively attending to the grating in the non-deprived eye substantially increased eye dominance but had little effect when the deprived eye was attended. This is likely to result from the strong starting bias in eye dominance of this observer (i.e. neutral PSE < 0). The relative dominance of one eye may have made it difficult for attentional mechanisms to push dominance further in the same direction.

At a group level, the results of a paired-sample t-test on the baseline measurements revealed that the absolute shift in PSE from the *neutral* condition (ΔPSE_neutral_), and the slope of the psychometric function, did not differ significantly between the two attentional states (left eye attended vs. right eye attended) (ΔPSE_neutral_: mean difference = − 0.004, 95% confidence interval (CI) = [− 0.32, 0.31], *t*_(6)_ = -0.03, *p* = 0.976, Cohen’s *d* = − 0.01; slope: mean difference = − 0.056, 95% CI = [− 0.39, 0.28], *t*_(6)_ = − 0.41, *p* = 0.694, Cohen’s* d* = − 0.16). Thus, the data were subsequently collapsed between these conditions by taking their average to evaluate the effect of attentional instructions. The results of a one-tailed, one-sample t-test revealed that when attentional instructions were provided, absolute ΔPSE_neutral_ was significantly greater than zero (*M* = 0.26, 95% CI = [0.14, Inf], *t*_(6)_ = 4.11, *p* = 0.003, Cohen’s* d* = 1.56). On the other hand, the slope of the psychometric function was not significantly altered relative to the neutral condition (see Fig. 5), as revealed by a paired-sample t-test (mean difference = 0.046, 95% CI = [− 0.13, 0.22], *t*_(6)_ = 0.65, *p* = 0.542, Cohen’s* d* = 0.24). Lastly, a paired-sample t-test showed a significant difference in the signed ΔPSE_neutral_ when the left eye, compared to the right eye, was attended (mean difference = 0.45, 95% CI = [0.09, 0.81], *t*_(6)_ = 3.08, *p* = 0.022, Cohen’s* d* = 1.16): the PSE was shifted in favour of the attended eye.

When no attentional instructions were provided, monocular deprivation shifted the psychometric function in favour of the previously deprived eye. To investigate if this change in performance was statistically significant at the group level, a linear mixed-effects model analysis was conducted. As not all participants completed exactly the same set of conditions, in terms of the eye(s) deprived and type of deprivation, the model included fixed effects of deprivation type (opaque vs. diffuse), deprived eye (left vs. right), and their interaction, and random effects of subject on the intercept and coefficients of the same variables. For the absolute shift in PSE relative to the baseline condition, the main effects of monocular deprivation type (*b* = 0.06, 95% CI = [− 0.05, 0.16], *F*_(1,11)_ = 1.39, *p* = 0.263) and eye (*b* = − 0.02, 95% CI = [− 0.09, 0.05], *F*_(1,11)_ = 0.46, *p* = 0.510) were not significant, nor was the interaction effect between the two variables (*b* = 0.07, 95% CI = [− 0.01, 0.15], *F*_(1,11)_ = 3.34, *p* = 0.095). For the slope of the psychometric function, similarly, neither the main effects (type: *b* = − 0.10, 95% CI = [− 0.28, 0.07], *F*_(1,11)_ = 1.71, *p* = 0.218; eye: *b* = 0.112, 95% CI = [− 0.13, 0.36], *F*_(1,11)_ = 1.12, *p* = 0.313), nor the interaction, were significant (*b* = − 0.11, 95% CI = [− 0.31, 0.09], *F*_(1,11)_ = 1.43, *p* = 0.257). Therefore, for subsequent analyses the data were collapsed first across eyes, and then between the two deprivation conditions, by taking the average. The results of a one-tailed, one-sample t-test revealed that absolute ΔPSE_baseline_ was significantly greater than zero after monocular deprivation (*M* = 0.22, 95% CI = [0.11, Inf], *t*_(6)_ = 3.85, *p* = 0.004, *d* = 1.46). However, monocular deprivation did not significantly alter the slope compared to baseline measurements (mean difference = − 0.047, 95% CI = [− 0.11, 0.02], *t*_(6)_ = − 1.71, *p* = 0.138, *d* = − 0.65).

We subsequently examined whether depriving a different eye had an impact on the direction (sign) of ΔPSE_baseline_ in the neutral condition. A linear mixed-effects model was constructed with a fixed effect of deprived eye (left vs. right), and random effects of subject $\times$ deprivation type on the intercept and coefficient. This analysis showed that depriving the left, compared to the right eye, produced significantly different signed ΔPSE_baseline_ (*b* = − 0.41, 95% CI = [− 0.71, − 0.10], *F*_(1,13)_ = 8.21, *p* = 0.013). That is, the PSE of the psychometric function was always shifted towards the previously deprived eye.

Having established separately the modulating effects of attentional instructions and monocular deprivation on sensory eye dominance, we next evaluated if the two processes interact. Figures 2 and 4 show that the effects of selective attention were evident not only in the baseline condition, but also after each form of monocular deprivation. Therefore, a linear mixed-effects model was first constructed to compare the absolute ΔPSE_neutral_ for the two deprivation type conditions. This model included a fixed effect of deprivation type (opaque vs. diffuser), and random effects of subject $\times$ deprived eye $\times$ attended eye on the intercept and coefficient. The results showed that overall the type of deprivation was not critical (*b* = 0.035, 95% CI = [− 0.10, 0.17], *F*_(1,28)_ = 0.29, *p* = 0.597), although one observer, S2, showed a marked difference between opaque patching and diffuser when the previously deprived eye was attended to (see Fig. 2).

To assess whether the presence of monocular deprivation altered the degree to which attentional instructions could modulate performance, the data were collapsed between the two types of monocular deprivation and then analysed using a linear mixed-effects model with a fixed effect of deprivation state (baseline vs. monocular deprivation), and random effects of subject $\times$ attended eye on the intercept and coefficient. This confirmed that monocular deprivation did not induce a significant change in the magnitude of ΔPSE_neutral_ induced by providing attentional instructions (*b* = 0.008, 95% CI = [− 0.10, 0.11], *F*_(1,26)_ = 0.02, *p* = 0.880). In other words, the modulation of eye dominance by attentional selection remained unaltered following monocular deprivation.

In summary, monocular deprivation shifted sensory eye dominance towards the previously deprived eye. Despite this deprivation-induced shift, the modulating effects of attention, directed towards the deprived or non-deprived eye, were still present and similar in magnitude to those observed in the baseline condition. Indeed, it is evident (e.g. Figure 4) that although deprivation shifted dominance in favour of the deprived eye, it could be completely counteracted if the non-deprived eye’s stimulus was selectively attended to.

## Discussion

Using an onset binocular rivalry task, where the inter-ocular contrast difference was varied, we have demonstrated independent modulation of sensory eye dominance by short-term monocular deprivation and concurrent attentional eye selection. The previously deprived eye, or the selectively attended eye, required less contrast to dominate perception during binocular rivalry. In most cases, the enhancement of the previously deprived eye could be counteracted if the participant was required to attend to the colour of the non-deprived eye’s grating. This shows that selective attention can quickly restore eye balance following monocular deprivation effects that require relatively prolonged periods to establish. Similarly, attending to the previously deprived eye added to the dominance of this eye beyond the facilitation induced by monocular deprivation. These results have important implications for understanding the mechanisms underlying plasticity of binocular interactions in the adult visual system. Although the rivalry task we used is subjective and might be prone to criterion effects^16^, we have taken care to randomise and counterbalance the conditions tested to mitigate any systematic biases in performance. Another concern for binocularly rivalry tasks relates to a potential stabilisation effect (the persistence of a perceptual configuration) due to the intermittent presentation of perceptually ambiguous stimuli^17^. However, it is unlikely to be the case in our experiments due to the randomisation procedures. The fact that we were able to shift the psychometric function, further supports the validity of using this task to assess sensory eye dominance. Given that there are inevitable individual differences present in the results, future research could be conducted to investigate the generality of these findings to the wider population.

The lateral shift of the contrast-response (psychometric) function is resonant of changes in effective contrast that follow neuronal adaptation in early visual cortex, where it has been proposed that a contrast gain control mechanism adjusts the output response to match prevailing levels in the environment^18–20^. A similar process of adjustment, mediated via contrast gain control, may also be at play in our experimental manipulations. Indeed, Zhou et al.^3^ have reported reciprocal changes in contrast gain in each eye following short-term monocular deprivation, characterised by a lateral shift of the monocular contrast sensitivity function. In the present study, we reveal effective contrast changes in one eye relative to the other by varying the inter-ocular contrast difference, while keeping the overall contrast level fixed. This suggests that inter-ocular gain control typically associated with the binocular combination of fusible stimuli^21^ also operates for rivalrous stimuli.

Attention is thought to play a key role in the perception of binocular rivalry. There are different ways this could be implemented: attention could act on the monocular signals from each eye^11^ or modify the relative weight of each eye’s input at binocular combination, via a process of mutual suppression or enhancement^22^. For tasks involving binocular rivalry, there is some existing evidence for the suppression of eye-based signals. For example, regions of the lateral geniculate nuclei (LGN) that display high degrees of ocular specificity, exhibit enhancement of functional magnetic resonance imaging (fMRI) signals for attended stimuli, and inhibition consistent with transient suppression of one image during rivalry^23,24^. Psychophysical evidence of eye-specific attentional modulation also comes from a modified binocular rivalry stimulus; gratings that were initially invisible had their contrast linearly ramped and the time taken to overcome the initial suppression was used as an index of the competitive strength of a target. Attending to monocular cues, without awareness of their eye of origin, significantly increased the strength of the cued stimulus^11^. Another study also reported modulations of binocular rivalry by endogenous attention and these were independent from modulations in pupil diameter^25^. This suggests that attention effects, rather than influencing the strength of the cued stimulus (indexed by changes in the pupil), may act during periods where inter-ocular competition is balanced. This result points to a difference between bottom-up and top-down influences on perceptual rivalry. However, laminar activation profiles in humans measured using ultra-high-field MRI have shown that spatial attention, directed towards a monocularly presented stimulus, increased V1 responses independently of eye-of-origin^26^. That is, voxels with a preference for either eye (identified by ocular dominance column mapping) increased activity by a similar amount. Nevertheless, the manipulation of attention, although combined with monocular stimulation, did not require stimulus selection between the two eyes (i.e. there was absence of inter-ocular conflict). Therefore, attentional modulation of monocular visual processing may be possible, but could require a competitive process, or behavioural need to select the input from one eye, to drive it.

At a neuronal level, selective spatial attention has been shown to alter the contrast or response gain of neurons throughout visual cortex. Specifically, the response (or sensitivity) to a stimulus presented at an attended location is strengthened compared to others at unattended locations^27,28^ and it has been proposed that these neuronal effects underpin enhanced behavioural performance for attended stimuli^29,30^. The attentional enhancement of neural responses is thought to operate proportionally across all stimulus contrast levels^31^. In monkeys performing spatial- or object-based attention tasks, the neural response function in V1 for supra-threshold targets is enhanced by a relatively constant amount, indicating an additive process that is largely invariant of stimulus contrast^32^. In humans, attention-based changes in contrast gain are particularly pronounced in V1 but occur throughout extra-striate visual areas^33^. If the effect of voluntary attention is first manifest at the level of the LGN, it may be further affected in cortex, presumably via a biased-competition framework^22,34^ that is more sensitive to properties of the stimulus itself. A feature of the biased-competition model of attention is that competitive interactions between stimuli are mutually suppressive and attention exerts its greatest effect where stimuli activate the same local region of cortex. The rivalrous oriented gratings used in the current study are well matched to the tuning properties of binocular cells in area V1 and attention may weight their responses in favour of the attended stimulus to resolve the conflict. This would set a new balance point at which relative contrast would tip perception from one input to the other.

Our data revealed that the effects of concurrent attentional eye selection and short-term monocular deprivation operate independently of each other. That is, enhancement in the dominance of the attended eye was quantitatively comparable before and after monocular deprivation. This implies that the two types of modulation may be driven by parallel mechanisms. Their outcomes might both end up in the ocular dominance columns of V1, such that the balance in the relative gain between the two eyes is modified. Recent work using laminar-resolved fMRI may shed important light on how this is implemented at a neuronal level. Using a visual task designed to induce concurrent bottom-up and top-down modulations, via manipulations of stimulus contrast and feature-based attention, respectively, Lawrence et al.^35^ found that BOLD responses were modulated in different layers of V1. Contrast-based modification was strongest in the middle (granular) cortical layers, whilst feature-based attention affected responses most prominently in superficial (agranular) layers. This layer specific modification of neural responses highlights that bottom-up and top-down influences have spatially distinct neural signatures, which alongside the discrete patterns of feedforward and feedback projections^36^ may represent distinct processing in each cortical pathway.

Our finding, of independent bottom-up and top-down contributions to the regulation of eye balance, has important implications for interventions designed to rebalance the two eyes in common developmental anomalies such as amblyopia. In amblyopia, an impediment to normal sensory development early in life produces an imbalance in spatial acuity between the eyes that usually prevents binocular fusion. Traditional therapy seeks to eliminate the monocular deficit, by patching the non-amblyopic eye for extended periods. More recently, the idea of inverse occlusion (patching the amblyopic eye) has gained prominence as a way of restoring inter-ocular balance^37,38^. There is also behavioural and electrophysiological evidence that selective attention is degraded for the amblyopic eye^39,^ ^40^ but see^41^ A deficit in attention may be an important, but largely unrecognised, component of the functional impairment in amblyopia and treatment directed towards it may help to either eliminate the inter-ocular imbalance, or supplement other approaches to visual rehabilitation^42^. Previous work using a “push–pull” protocol and manipulation of exogenous attention has shown promise in reducing sensory eye imbalance in both healthy and amblyopic individuals^43,44^. Based on the current findings, extending this training protocol to endogenous attentional eye selection, while leveraging the independent effects of monocular deprivation, may help improve amblyopia treatment effectiveness.

## Data Availability

The datasets generated during and/or analysed during the current study are available from the corresponding author on reasonable request.
